# Supplementary figures and images for: Optogenetic activation of spinal microglia triggers chronic pain in mice
Source: PLoS Biol. 2021 Mar 19;19(3):e3001154. doi: 10.1371/journal.pbio.3001154 (PMC8011727; doi:10.1371/journal.pbio.3001154)

Figure 6C

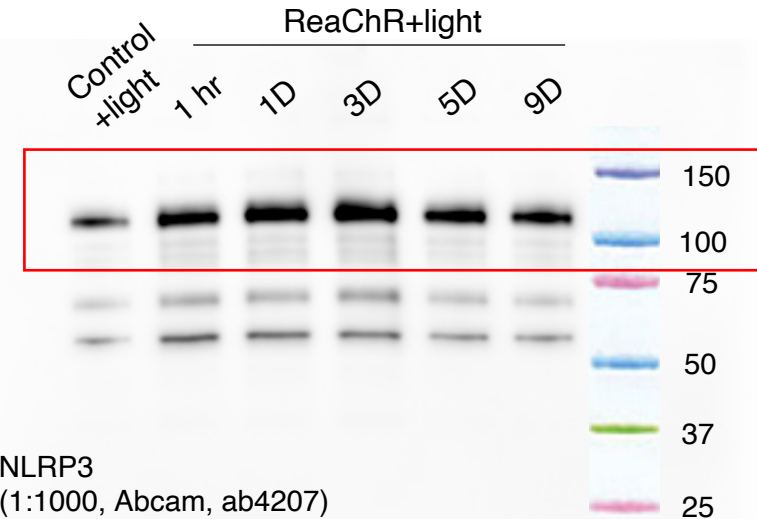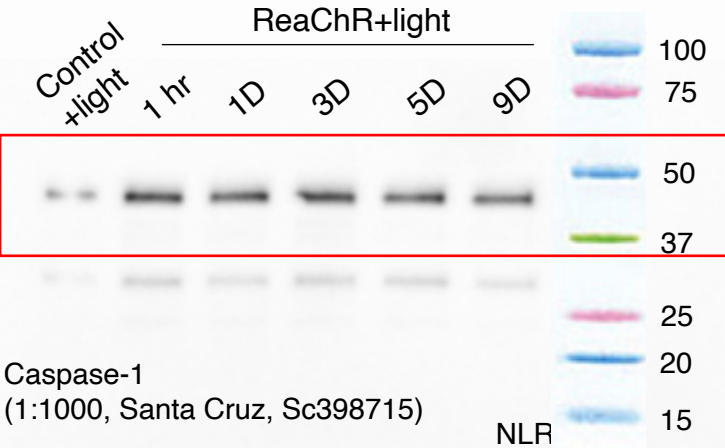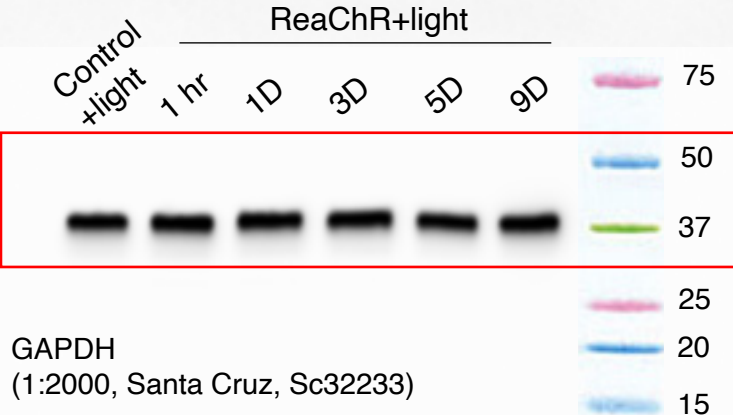

Supplementary Figure 7

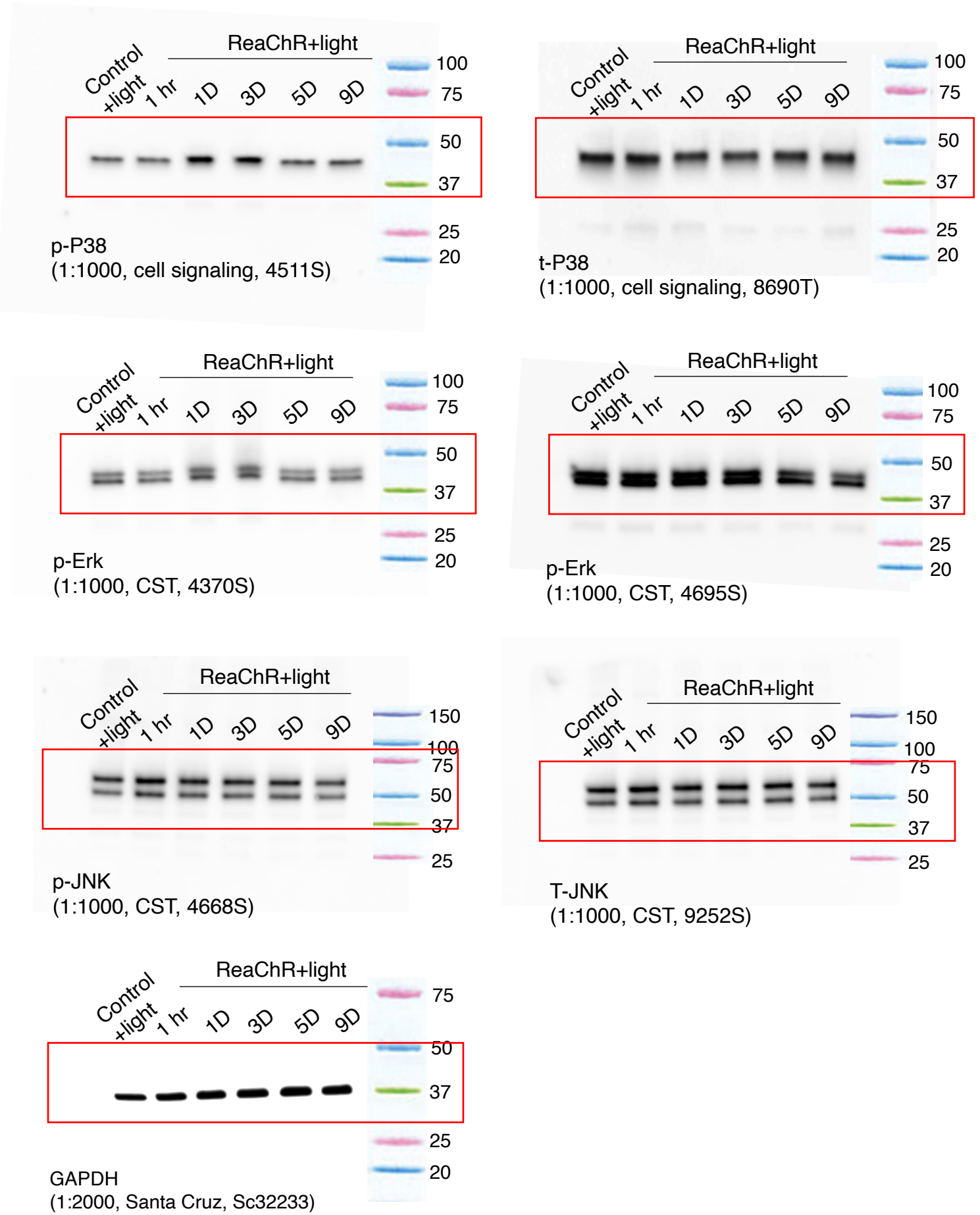

Supplementary Figure 7

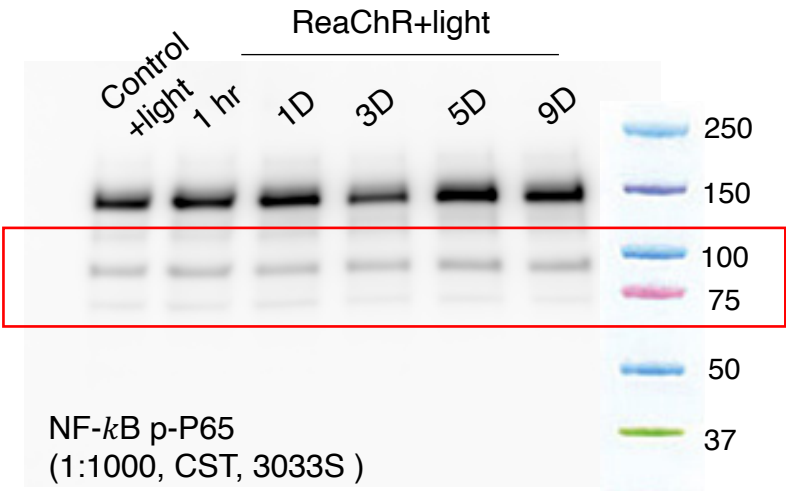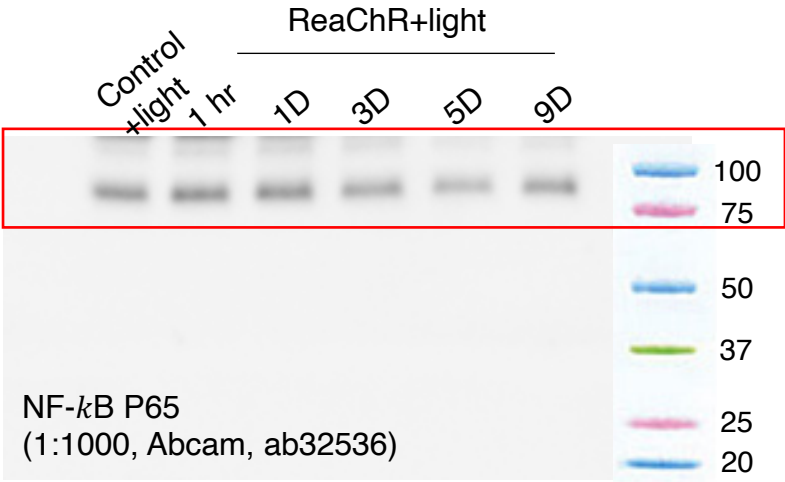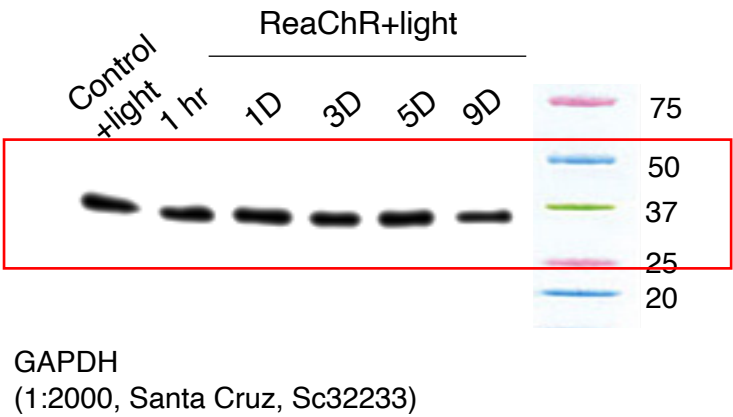

Supplement: S1 Raw Images — (PDF) [file pbio.3001154.s002.pdf]

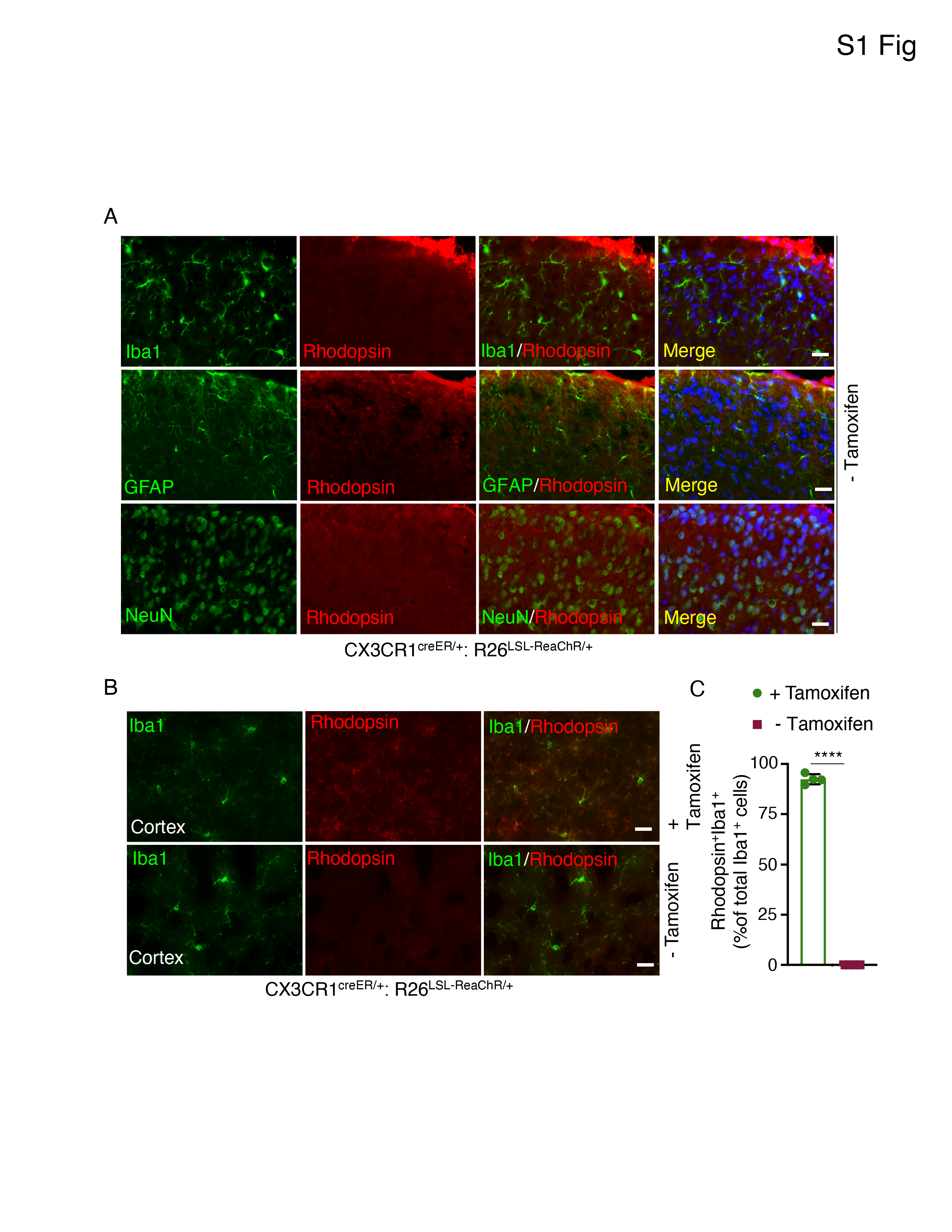

Supplement: S1 Fig — (A) Representative immunostaining images of rhodopsin (red) with either Iba1, GFAP, or NeuN (green) in the spinal dorsal horn of CX3CR1creER/+: R26LSL-ReaChR/+ mice without TM injection. Rhodopsin expression was not detected under these conditions. Scale bar, 40 μm. (B) Representative immunostaining images of rhodopsin with Iba1 in the cortex of CX3CRcreER/+: R26LSL-ReaChR/+ mice with or without TM injection. Rhodopsin expression was co-localized with Iba1+ cells in the cortex of CX3CRcreER/+: R26LSL-ReaChR/+ mice when TM was administered but was absent without TM injection. Scale bar, 40 μm. (C) Summarized data showing the co-localization of Rhodopsin with Iba1+ cells. Data are presented as mean ± SEM, n = 4 mice/group, ****P < 0.0001. Unpaired Student t test. For data plotted in graphs, see S1 Data. ReaChR, red-activated channelrhodopsin; TM, tamoxifen. (TIF) [file pbio.3001154.s003.tif]

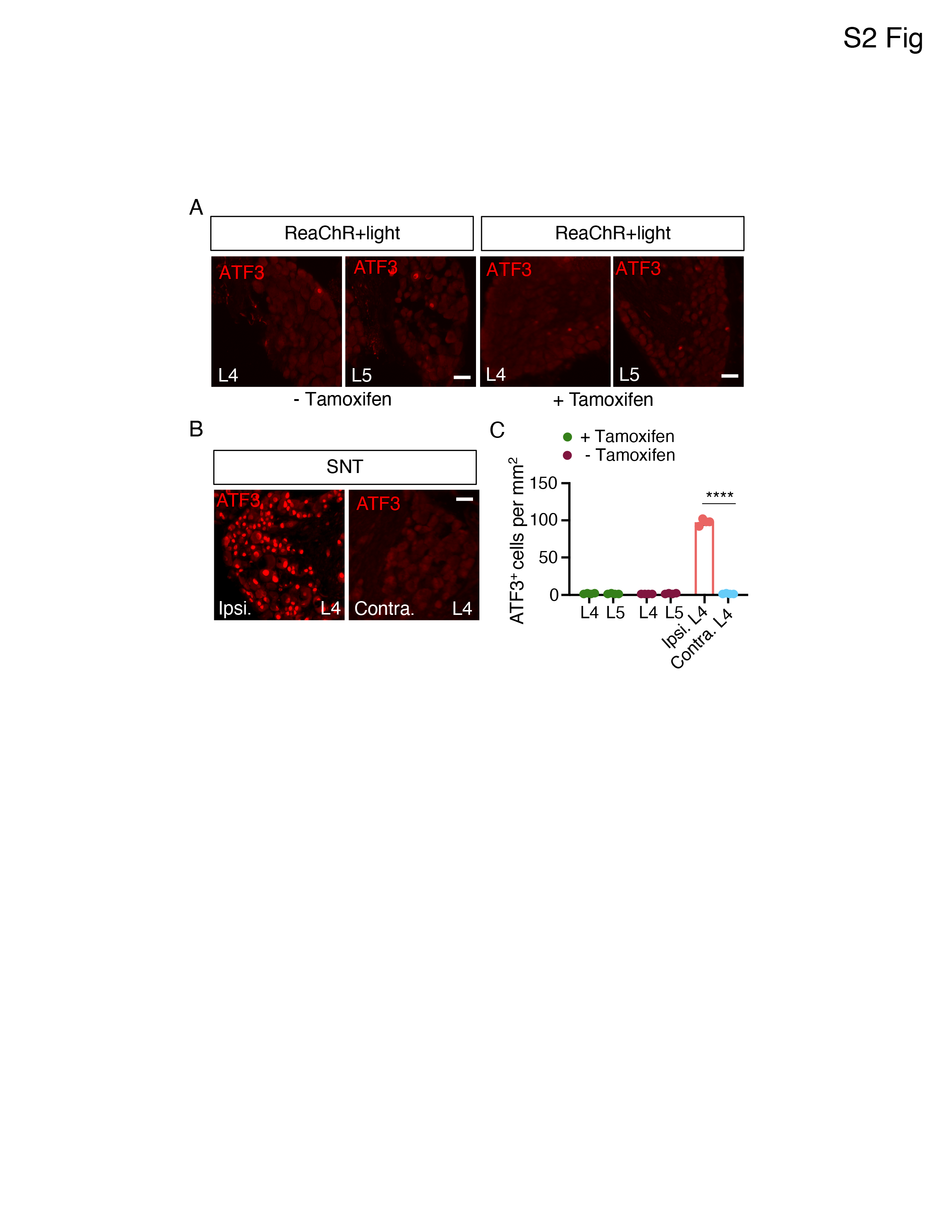

Supplement: S2 Fig — (A) Representative immunostaining images of ATF3 in L4-5 DRGs of ReaChR mice following light stimulation. Very few ATF3+ cells were observed following stimulation. Scale bar, 40 μm. (B) SNT surgery induced significant ATF3 expression within ipsilateral L4 DRG (Ipsi.) but not contralateral side (Contra.). Scale bar, 40 μm. (C) Summarized data showing the ATF3+ cells. n = 4 mice/group, ****P < 0.0001. Two-way ANOVA with multiple comparisons. For data plotted in graphs, see S1 Data. DRG, dorsal root ganglion; ReaChR, red-activated channelrhodopsin; SNT, spinal nerve transection. (TIF) [file pbio.3001154.s004.tif]

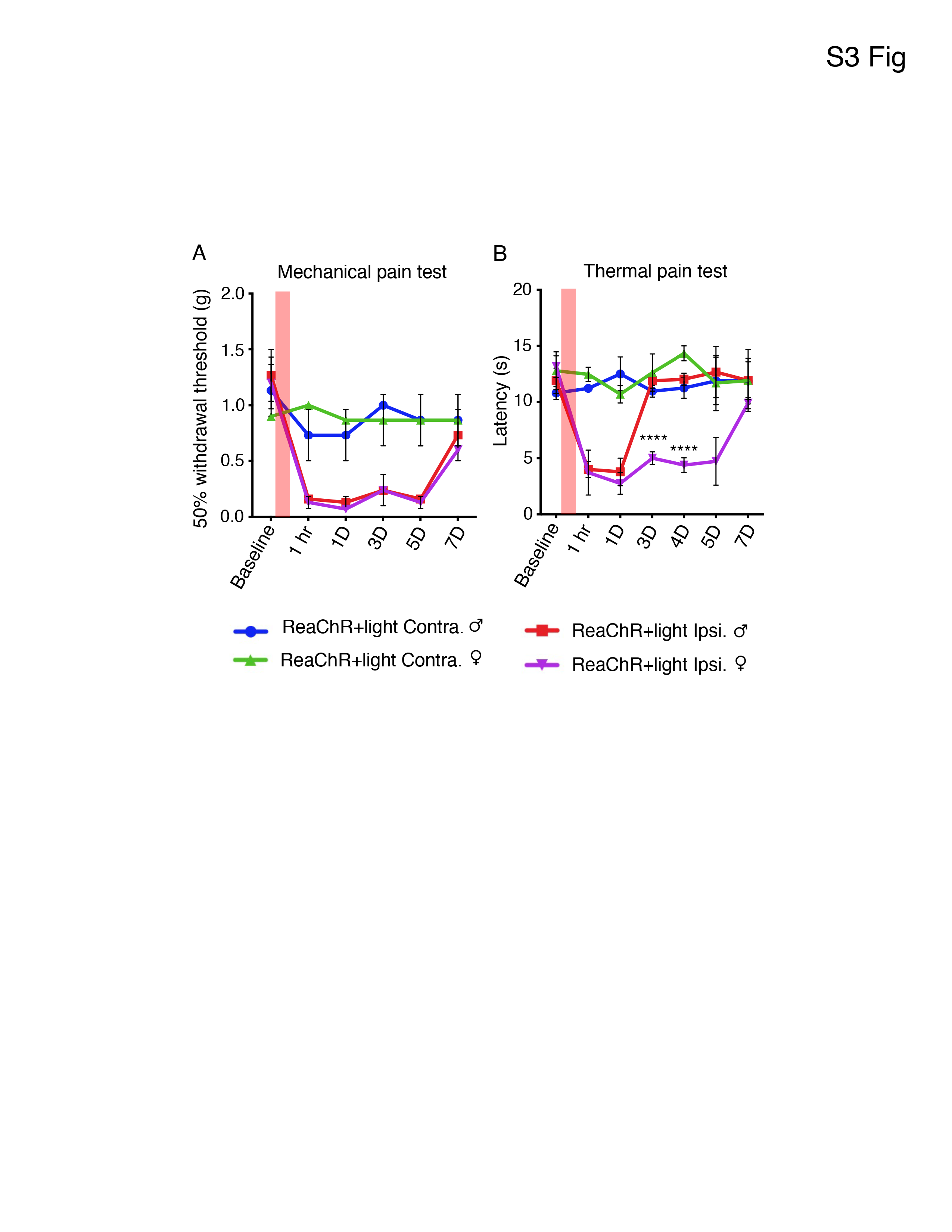

Supplement: S3 Fig — (A, B) Measurement of mechanical (A) and thermal (B) pain hypersensitivity in male and female ReaChR mice. Results show that no difference was observed between male and female mice in regard to mechanical allodynia. However, thermal hypersensitivity was observed to last longer in female mice when compared to male mice. Data are presented as mean ± SEM, n = 5 mice/group ****P < 0.0001, male vs. female. Two-way ANOVA with multiple comparisons. For data plotted in graphs, see S1 Data. ReaChR, red-activated channelrhodopsin. (TIF) [file pbio.3001154.s005.tif]

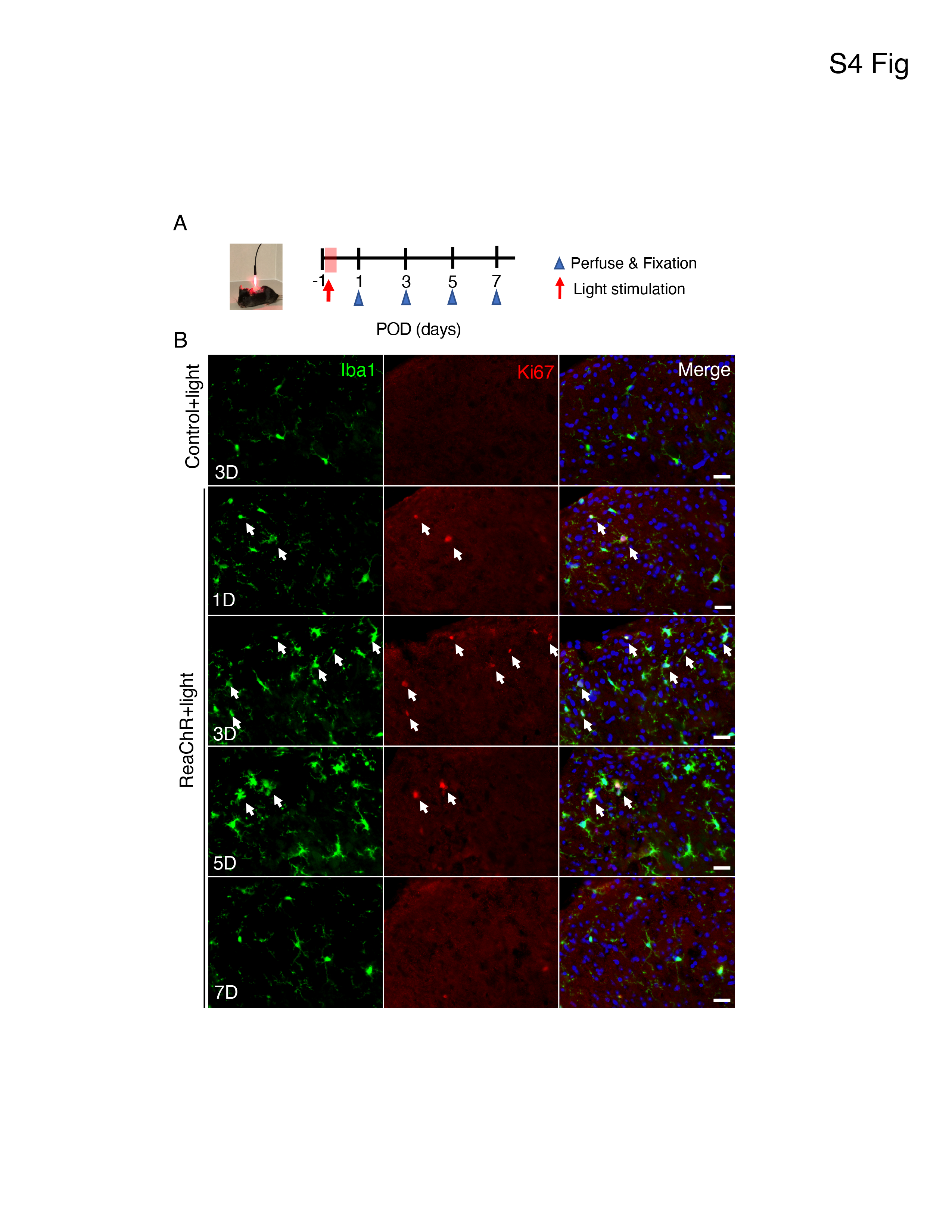

Supplement: S4 Fig — (A) Timeline of experimental procedures. (B) Immunofluorescence images showing co-localization of Ki67 (red) and Iba1 (green) at 1, 3, 5, and 7 days after optogenetic stimulation compared with control group. Arrows indicate the Ki67+Iba1+ cells. Scale bar, 40 μm. (TIF) [file pbio.3001154.s006.tif]

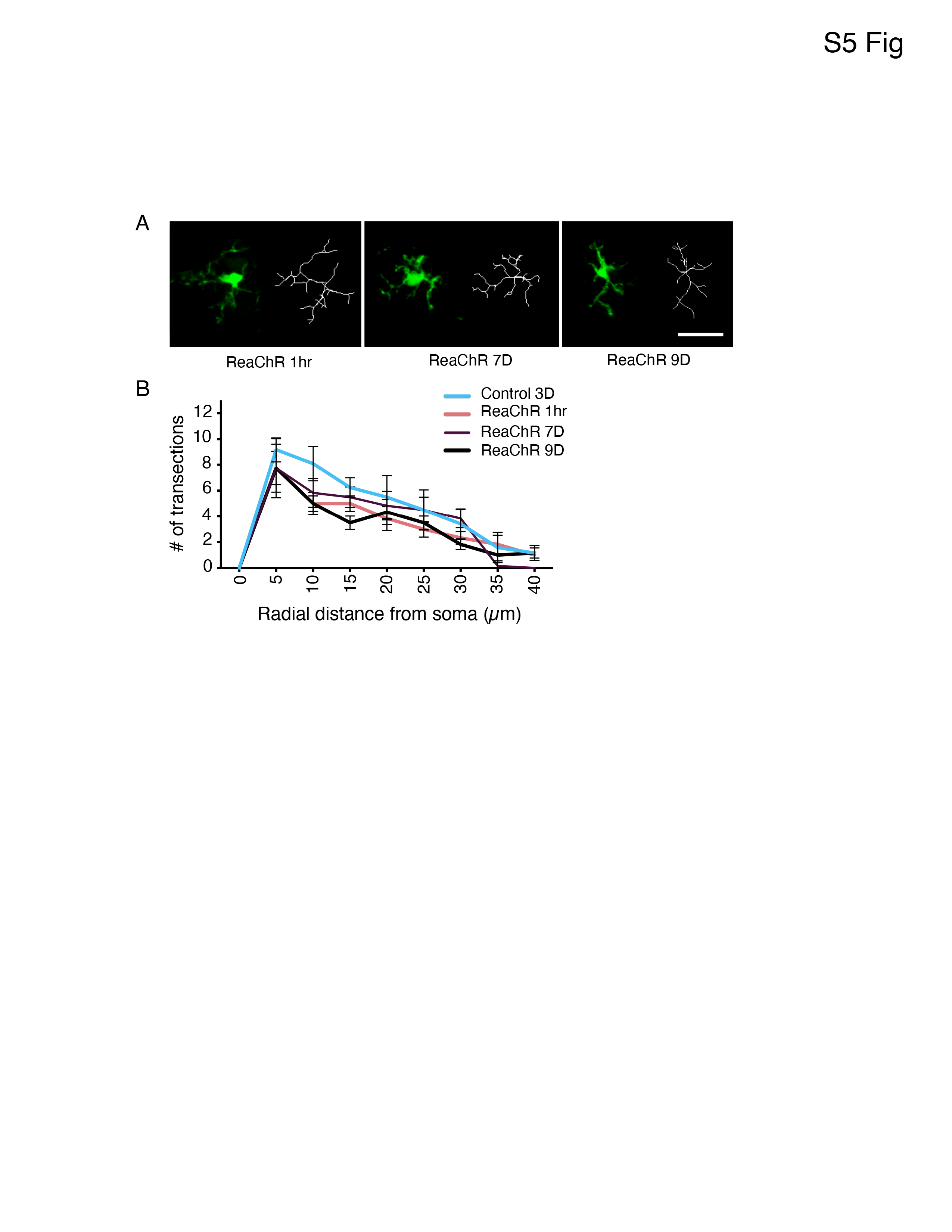

Supplement: S5 Fig — (A) Representative images of spinal cord microglia immunostained by Iba1 and skeletonized structure following optogenetic stimulation. Scale bar, 40 μm. (B) Summarized Sholl analysis data showing no significant change in the complexity of ReaChR microglia at 1 h, or 7 and 9 days after light stimulation compared with the control group. Data are presented as mean ± SEM, n = 5 mice/group, Two-way ANOVA. For data plotted in graphs, see S1 Data. ReaChR, red-activated channelrhodopsin. (TIF) [file pbio.3001154.s007.tif]

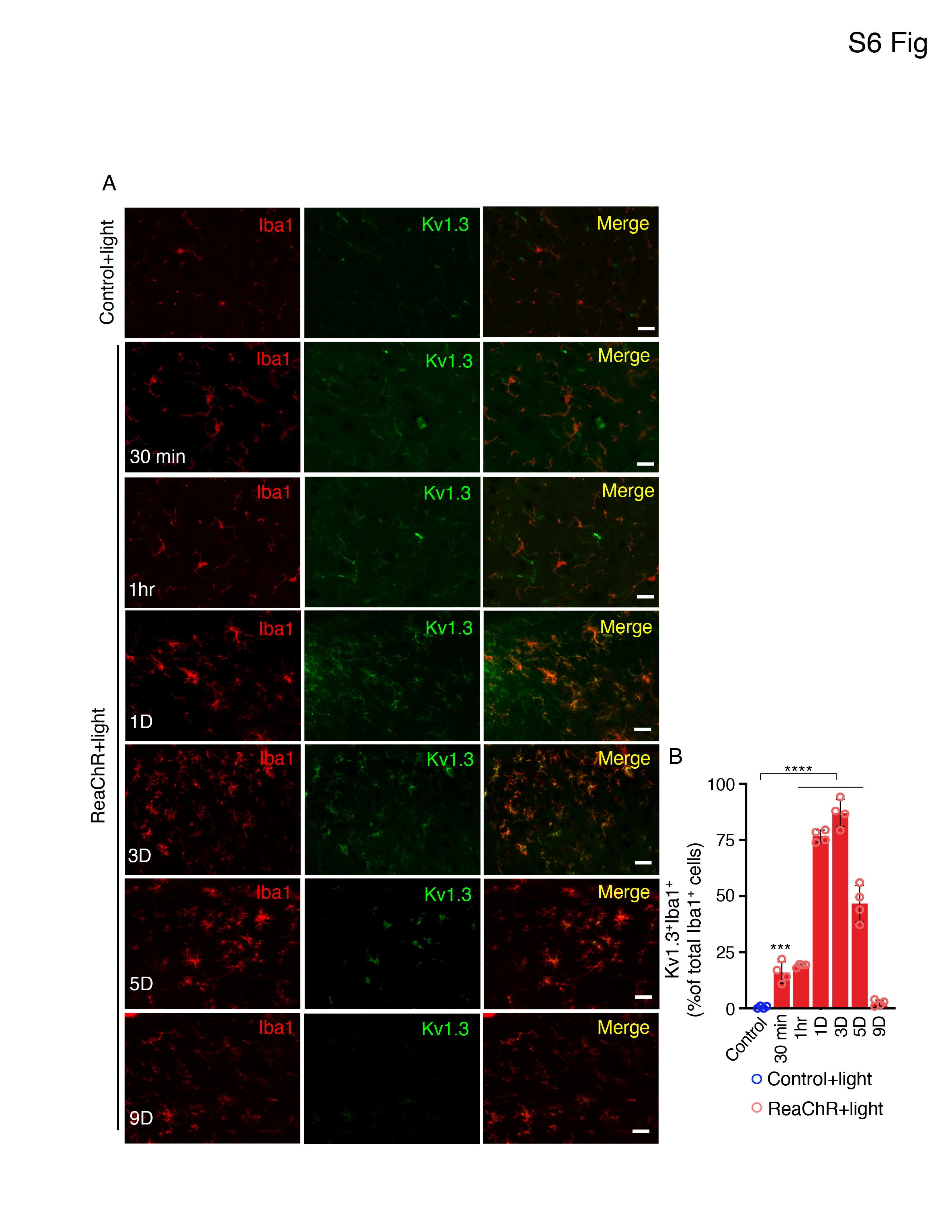

Supplement: S6 Fig — (A) Representative immunostaining images showing expression of Kv1.3 (green) in Iba1+ (red) microglia at 30 min, 1 h, 1, 3, 5, and 9 days after light stimulation in the ipsilateral dorsal horn. Results indicated Kv1.3 expression increased gradually from 30 min to 3 days after light stimulation in ReaChR mice as compared to control group. Scale bar, 40 μm. (B) Summarized data showing the co-localization of Kv1.3 with Iba1+ cells. Data are presented as mean ± SEM, n = 4 mice/group, ***P < 0.001, ****P < 0.0001. One-way ANOVA with multi-comparisons. For data plotted in graphs, see S1 Data. ReaChR, red-activated channelrhodopsin. (TIF) [file pbio.3001154.s008.tif]

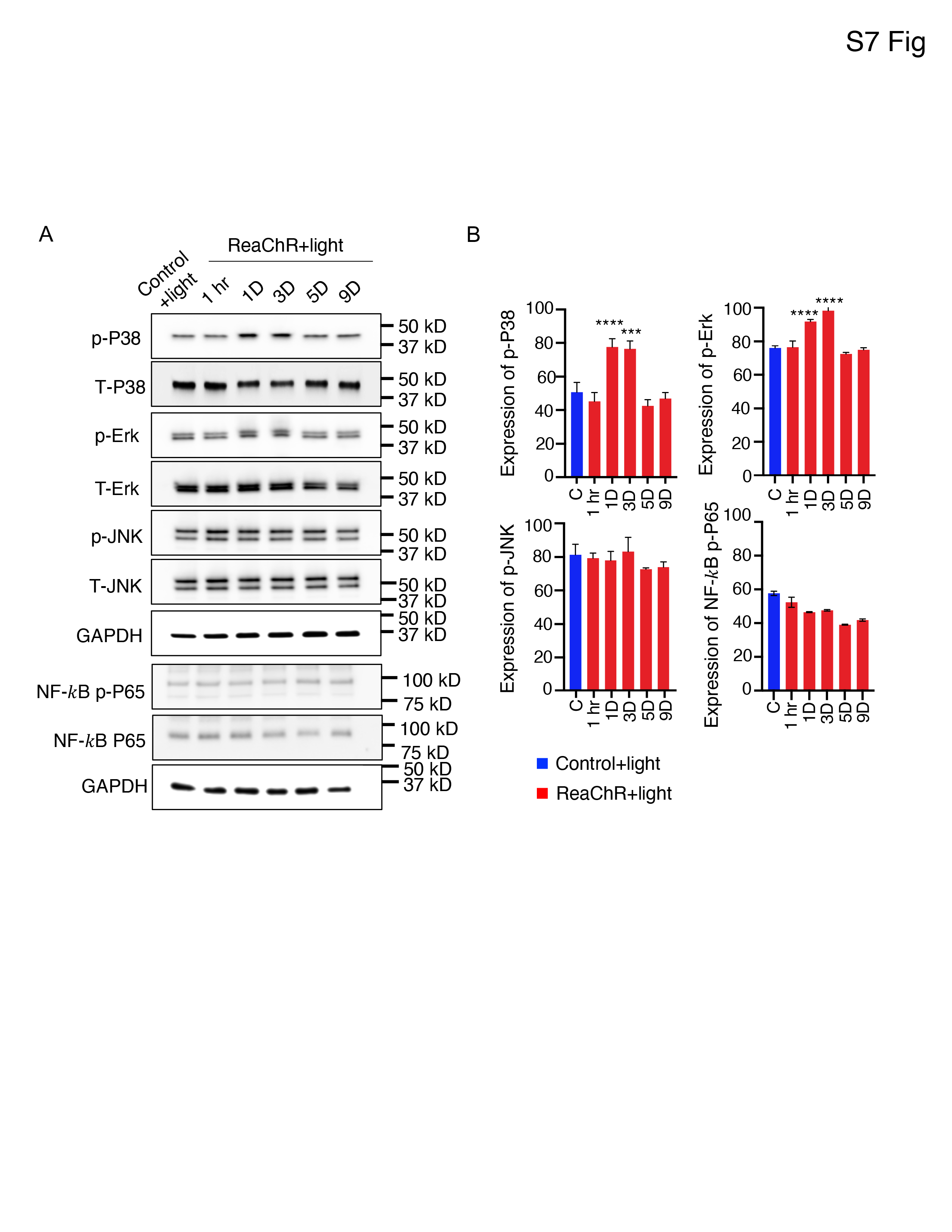

Supplement: S7 Fig — (A, B) Representative western blot images (A) and quantification data (B) showing expression of MAPK signaling molecules (P38, Erk, and JNK) and NF-κB p-P65 in L4-5 level of the dorsal horn after optogenetic stimulation. Up-regulated expression of P-p38 and P-Erk was observed at 1 to 3 days in the ipsilateral spinal dorsal horn compared control mice after light stimulation. However, no significant difference in P-JNK and NF-κB p-P65 was observed. Data are presented as mean ± SEM, n = 4–6 mice/group, GAPDH was used as internal control. ***P < 0.001, ****P < 0.0001. One-way ANOVA with multi-comparisons. For data plotted in graphs, see S1 Data. Uncropped western blot images were included in the Supporting information file. ReaChR, red-activated channelrhodopsin. (TIF) [file pbio.3001154.s009.tif]

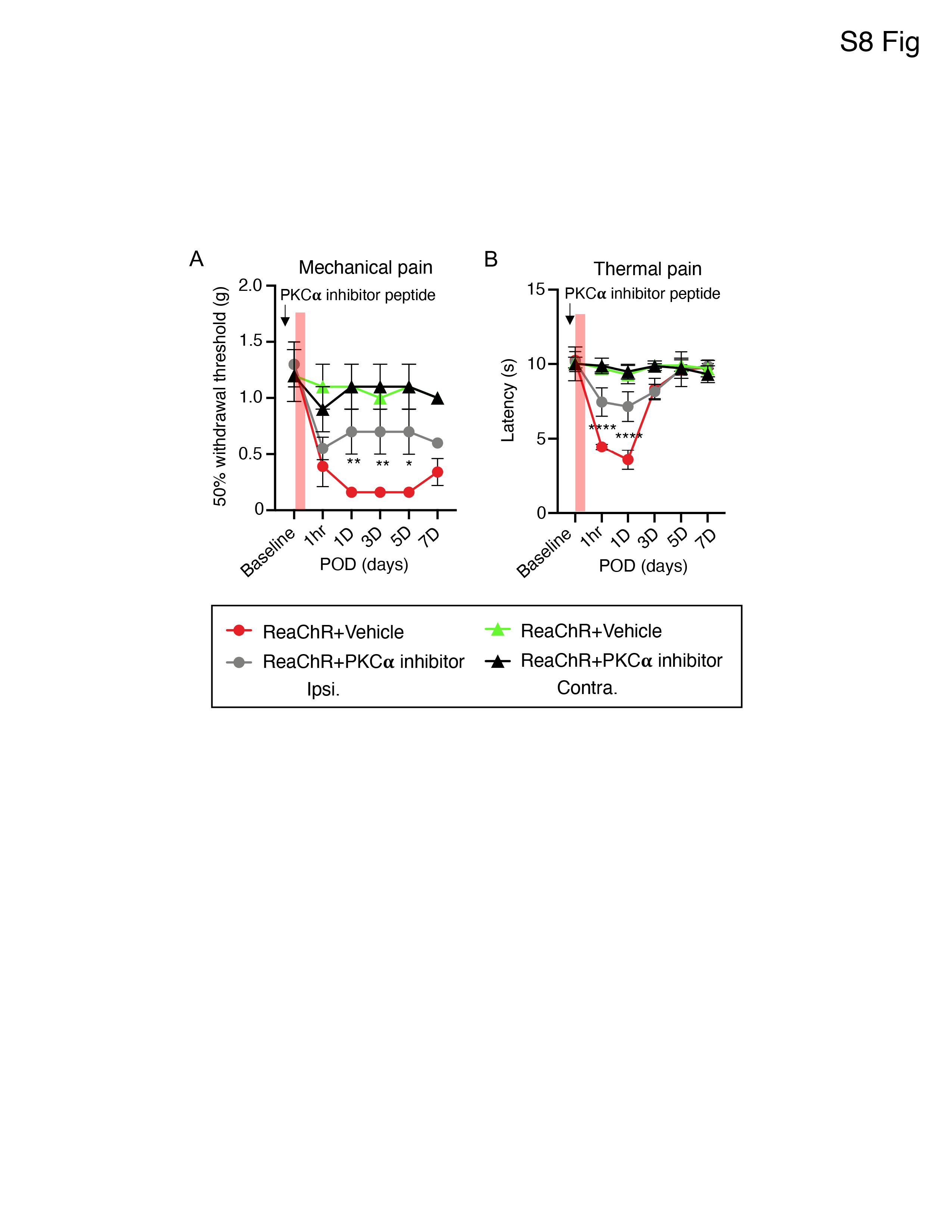

Supplement: S8 Fig — (A, B) Light-induced mechanical (A) and thermal (B) pain hypersensitivity in ipsilateral or contralateral side of ReaChR mice after treatment of PKCα inhibitor peptide or vehicle. Data represented as mean ± SEM, n = 4 mice/group. ****P < 0.0001, **P < 0.01, *P < 0.05 vehicle vs. PKCα inhibitor. Two-way ANOVA with multi-comparisons. For data plotted in graphs, see S1 Data. ReaChR, red-activated channelrhodopsin. (TIF) [file pbio.3001154.s010.tif]

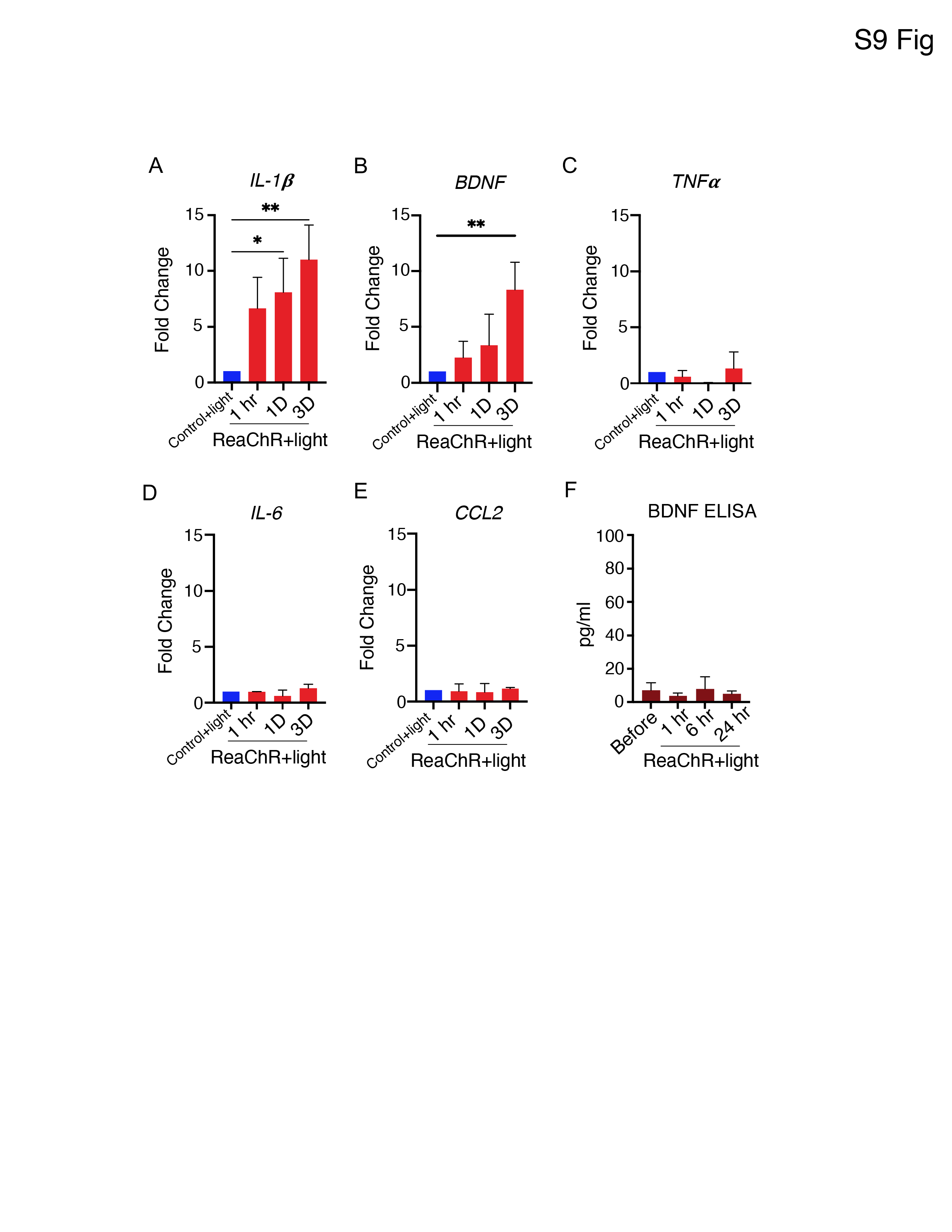

Supplement: S9 Fig — (A-E) Real-time PCR analysis data showing mRNA expression fold change of IL-1β (A), BDNF (B), TNFα (C), IL-6 (D), and CCL2 (E), in the L4-5 level of the dorsal horn after optogenetic stimulation. GAPDH was used as a reference control to normalize the expression level of the target gene (ΔCT) to correct for experimental variation. Relative mRNA levels were calculated according to the 2−ΔΔCT method. (F) Pooled ELISA results showing no significant change of BDNF in culture media from primary microglia following optogenetic stimulation. *P < 0.05, **P < 0.01. One-way ANOVA with multi-comparisons. For data plotted in graphs, see S1 Data. IL, interleukin. (TIF) [file pbio.3001154.s011.tif]

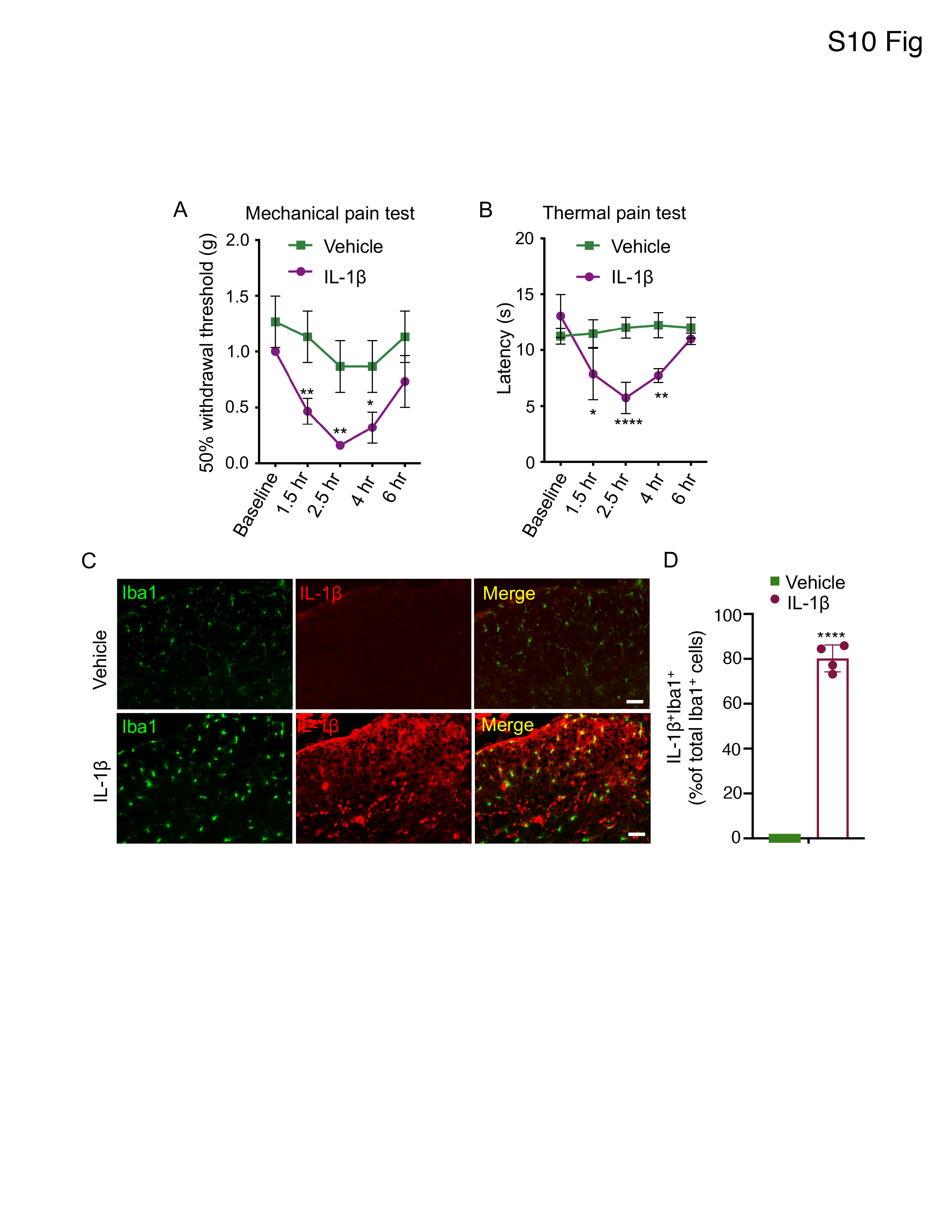

Supplement: S10 Fig — (A, B) Measurement of mechanical (A) and thermal (B) pain hypersensitivity of control mice after i.t. injection of recombinant IL-1β (10 μL, 20 ng/mL). Results show enhanced mechanical and thermal hypersensitivity following administration of IL-1β. n = 5 mice/group *P < 0.05, **P < 0.01, ****P < 0.0001, Vehicle vs. IL-1β. Two-way ANOVA with multi-comparisons. (C) Representative immunostaining images of IL-1β (red) expression in Iba1+ (green) cells within the spinal dorsal horn1 day after vehicle/IL-1β i.t. injection. IL-1β expression in Iba1+ microglia was increased in exogenous IL-1β (i.t.) injection group, whereas no expression was observed in vehicle treated group. Scale bar, 40 μm. (D) Summarized data showing the co-localization of Kv1.3 with Iba1+ cells. Data are presented as mean ± SEM, n = 4 mice/group, ****P < 0.0001, unpaired Student t test. For data plotted in graphs, see S1 Data. IL, interleukin; i.t., intrathecal. (TIF) [file pbio.3001154.s012.tif]

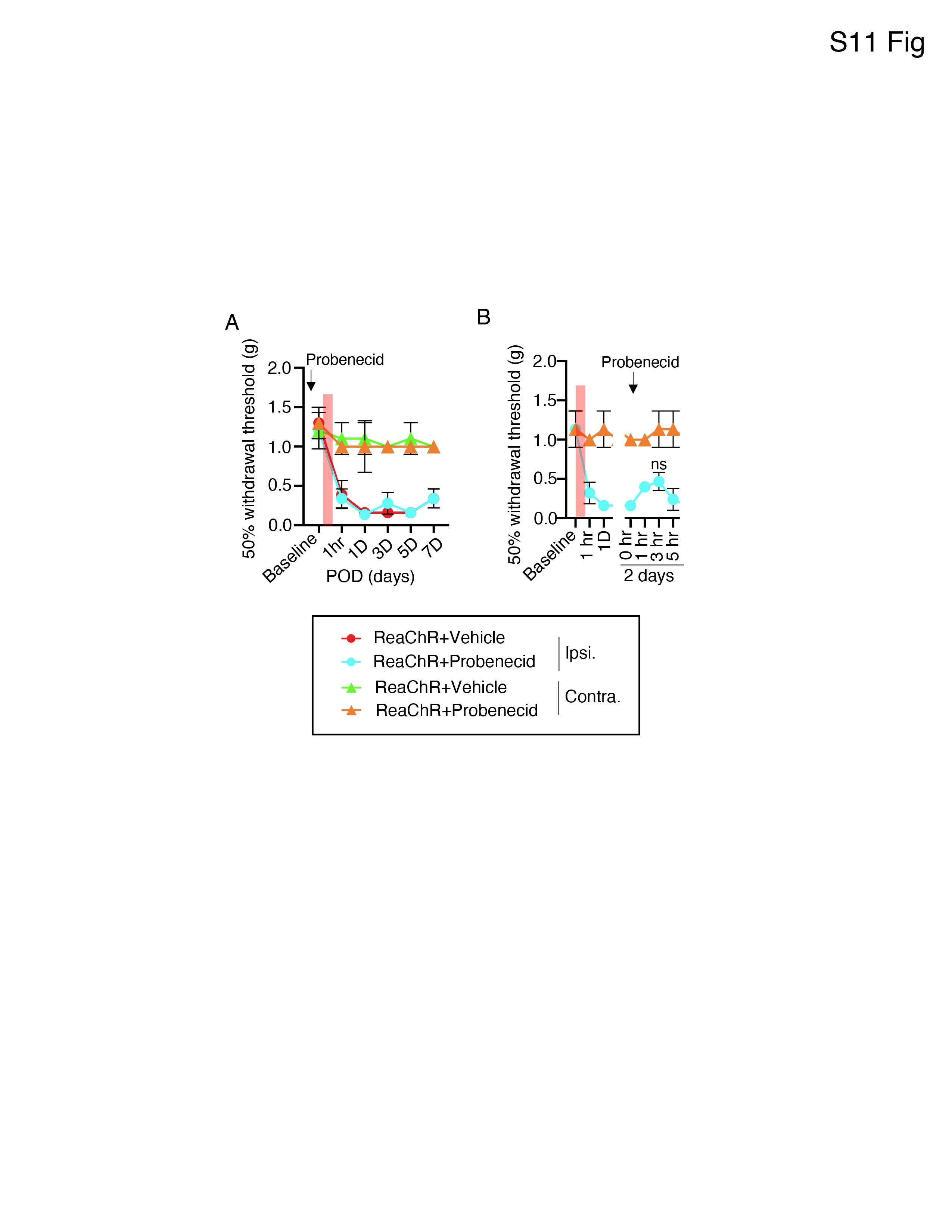

Supplement: S11 Fig — (A) Single systemic injection of probenecid (100 mg/kg) before optic stimulation did not prevent the progressive reduction in mechanical threshold. (B) Probenecid administration at 2 days after optic stimulation did not attenuate the established mechanical allodynia following microglial optogenetic activation. Data represented as mean ± SEM, n = 4 mice/group. Two-way ANOVA with multi-comparisons. For data plotted in graphs, see S1 Data. ReaChR, red-activated channelrhodopsin. (TIF) [file pbio.3001154.s013.tif]

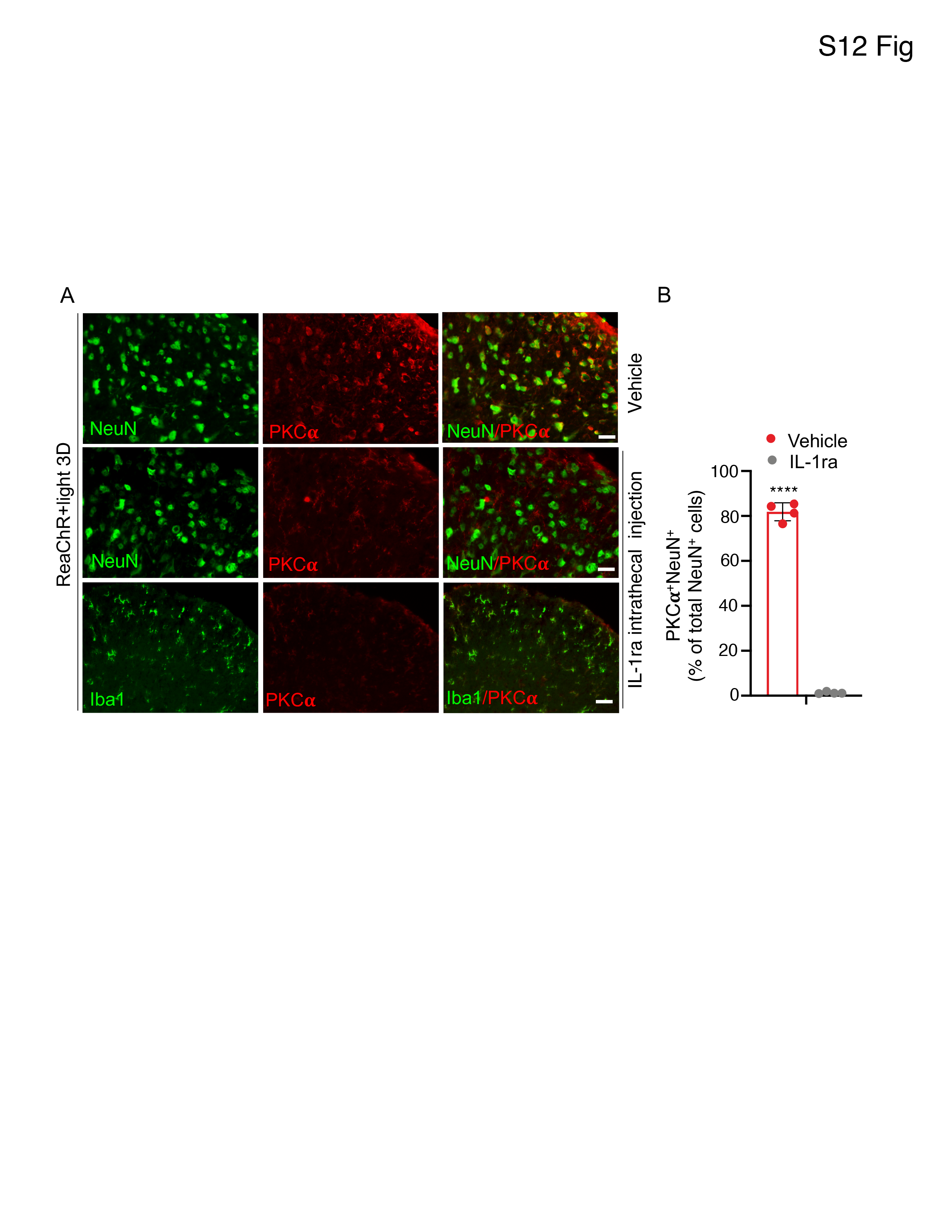

Supplement: S12 Fig — (A) Representative images of PKCα immunostaining (red) with NeuN (green) in the spinal dorsal horn of ReaChR mice following i.t. injection of vehicle or IL-1ra (10 μL, 50 ng/mL). PKCα expression was not detected in NeuN+ cells receiving IL-1ra 3 days after light stimulation compared with vehicle group. Scale bar, 40 μm. (B) Summarized data showing the co-localization of PKCα with NeuN+ cells. Data are presented as mean ± SEM, n = 4 mice/group, ****P < 0.0001, unpaired Student t test. For data plotted in graphs, see S1 Data. IL, interleukin; IL-1ra, IL-1 receptor antagonist; i.t., intrathecal; ReaChR, red-activated channelrhodopsin. (TIF) [file pbio.3001154.s014.tif]
