# Supplementary material for: Optogenetic activation of spinal microglia triggers chronic pain in mice
Source: PLoS Biol. 2021 Mar 19;19(3):e3001154. doi: 10.1371/journal.pbio.3001154 (PMC8011727; doi:10.1371/journal.pbio.3001154)
Supplement: Attachment — Submitted filename: Response to reviewers 20210124.pdf [file pbio.3001154.s015.pdf]

Point-to-point response to the reviewers' comments (PBIOLOGY-D-20-02660R1)

Responses to Reviewer 1:

In this manuscript Yi et al nicely demonstrates that 30-min in vivo optogenetic activation of spinal cord microglia leads to increased microglial IL-1b production, enhanced C-fiber responses and spinal cord neuron activities, resulting in mechanical and thermal hypersensitivities that last for several days. The experiments are well designed, and the results are overall convincing. However, I have the following minor concerns.
Response: We appreciate the reviewer's positive feedback.

Minor points:

1. As the authors point out in the Discussion, optogenetic activation may not represent the physiological function of microglia. The fact that a 30-min optogenetic stimulation results in super-long pain behaviors also suggests that the stimulation is probably unnaturally strong. As the result, the authors need to clarify in the Discussion that although optogenetic activation of microglia can lead to enhanced neuronal activity and pain behavior, its application in understanding microglia function in nature chronic pain conditions might be limited.
Response: We agree with the reviewer that optogenetic stimulation of microglia is probably unnaturally strong. Therefore, there were limitations to use optogenetic approaches for studying microglia function in nature chronic pain conditions. We have added the discussion in the revised manuscript (**Please see paragraph 1, page 14**).

2. In Introduction, a citation should be added to support the claim that "Purinergic signaling can directly activate ionotropic P2X4 and P2X7 receptors that are highly calcium permeable".
Response: Yes, we now added citation in Introduction (**please see paragraph 2, page 3**).

3. It is unclear what tests were performed in Fig. 3E-F.
Response: We performed mechanical and thermal pain tests with different duration of light stimulation (5, 15, and 30 min) in **Figure 3E-F**. We found that mechanical and thermal pain were increased at one hour after 30-min light stimulation on the ipsilateral side compared to contralateral side. We now added designation of pain tests and pairwise comparisons in **Figure 3E-F**.

4. It would be interesting to know is optogenetic activation can induce microglia proliferation.
Response: We thank the reviewer for the great suggestion. Accordingly, we performed additional experiments using Ki67 immunostaining co-labeled with Iba-1 to study microglial proliferation after optogenetic stimulation. We found that there is indeed increased proliferation in Iba1^{+} microglia at 3 days and then the proliferation was reduced at 5-7 days in ReaChR mice after optogenetic stimulation. We included additional data in the **Figure 4C** and **Supplementary Figure 4A-B** (**Please also see paragraph 1, page 7**).

5. There is no control in Fig. S4C.
Response: Sorry for the negligence. We now added control (Vehicle treatment group) in Fig. S4C (**now Supplementary Figure 12B**).

6. As the authors point out, there is no direct evidence suggesting the increased intracellular Ca2+ after optogenetic stimulation of microglia, they probably should tune down the function of ReaChR-induced Ca2+ in Discussion.

Response: Yes, we understand the reviewer's concern. We included a few more points related to ReaChR2 induced Ca^{2+}: (1) ReaChR is known as non-selective cation channels (Lin et al., 2013). In addition, ReaChR activation in neurons induces Ca^{2+} elevation (Chen et al., 2019). (2) To examine the potential role of Ca^{2+} influx after ReaChR activation, we performed additional experiments to chelate extracellular Ca^{2+} using EGTA (5 mM) during light stimulation *in vitro*. We observed that ReaChR-dependent IL-1β release was inhibited by chelating extracellular Ca^{2+}. Together, we believe that the increased intracellular Ca^{2+} is a potential consequence after optogenetic stimulation of microglial ReaChR. We included additional data **in the Figure 6H** and revised the manuscript in result part (**Please see paragraph 3, page 9**).

7. In the "Optogenetic ferrule implantation and light stimulation" part of Materials and Methods, "craniotomy" should not be the word to describe the procedure because it is the procedure to remove part of the skull bone for exposing brain. In the same section it is unclear how the authors can ensure the dura membrane to be intact.

Response: We thank the reviewer for the comment. We corrected "craniotomy" into "A small laminectomy" in Materials and Methods (**Please see page 15**). Also, we added more descriptions for an optogenetic delivery system designed to prevent damage to the dura. First, we made a small hole of spinal cord vertebra by using dental drill. Second, taking advantage of the shape of the ferrule having a hollow space, the optic fiber inserted into the ferrule was exposed at hollow space inside of the part facing down of ferrule (**Reviewer Figure 1**). Third, by making the lateral length of the ferrule equal to the length of the optic fiber to the bottom (the exposed optic fiber is within 0.3 mm), it is possible to access the small hole without damaging the dura membrane. The descriptions were added in the materials and methods part (**Please see Materials and Methods, page 15**).

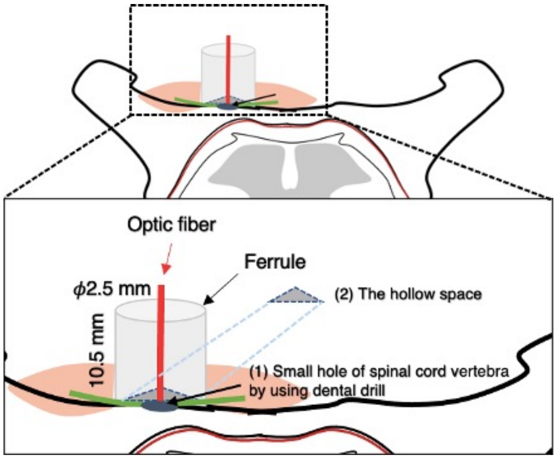

Reviewer Figure 1. A schematic diagram of the optogenetic ferrule placement for light delivery to the spinal cord (1) A small laminectomy of spinal cord vertebra by using dental drill and (2) optic fiber inserted into the ferrule was exposed at hollow space inside of the part facing down of ferrule.

8. In Fig. 3G, 3H, 7C, 7D and S3, is it true that the optogenetic stimulation of microglia occurred right before the baseline tests?

Response: Sorry for the confusion. We actually tested baseline right before light stimulation. We now corrected the color bar of light stimulation to reflect the actual experimental paradigm in the figures.

<u>Responses to Reviewer 2:</u>

This manuscript examines consequences of activating spinal microglia via optogenetic methods on pain behaviors in mice. The authors show that activated microglia trigger long lasting hypersensitivity that is mediated via an IL1beta dependent activation of C-fiber inputs. The use of optogenetics to activate microglia is a very nice approach (despite the caveats stated by the authors), however, I am not so sure that the actual findings are all that novel. It has been shown by others that spinal injection of ATP activated microglia mediates a rapid pain response. It has also been shown by others that IL1beta is produced by microglia and that this causes pain responses - therefore I do not see a large conceptual advance here other than to say that the authors are able to acutely activate microglia and get a response in vivo (which is cool). This work may be better suited as a technical report.
Response: We thank the reviewer's feedback on our study. Appreciate that the reviewer agrees optogenetic tool is a cool approach to manipulate microglia in high spatiotemporal precision. Although spinal microglia are known as a key player in pain, there is limited direct evidence showing selective microglial activation *in vivo* is sufficient to induce chronic pain.

1. I am not sure that the characterization of the ReaCHr2 current needs to be in a full main figure - it seems to me that this is mostly a control to show that the construct is functionally expressed. This could thus be moved to a supplementary figure.
Response: Since this is the first time to demonstrate that optogenetic approach is working in microglia, we would like to keep the electrophysiological results in the main figure. In addition, the results lay the foundation for the rest of behavioral studies after optogenetic stimulation of spinal cord microglia *in vivo*.

2. It seems to me that Fig 4 should precede Figure 3 in terms of logical flow - one would normally first demonstrate that light in facts activates the microglia, prior to doing behavioral measurements
Response: We thank the reviewer for the suggestion. Although some studies like to present microglial activation first before the behavioral experiments, the logical flow in our current study is as following: We first demonstrated the ReaChR is expressed in microglia (**Figure 1**) and is functionally working to mediate inward current and depolarization upon light stimulation (**Figure 2**). We next asked the question whether microglial ReaChR activation is important for pain behaviors, and we found selective microglia activation is sufficient to trigger chronic pain (**Figure 3**). In Figure 4-7, we went on to reveal the cellular (**Figure 4-5**) and molecular mechanisms (**Figure 6-7**) of microglial ReaChR-induced chronic pain. We hope this could clarify the study design and the reviewer would kindly agree with the logical flow we presented in this study.

3. Some form of quantification should be provided for Fig 5F and G. The authors in fact state that they saw a significant increase in PKC expression, but I wonder whether this is semantics, or whether there was in fact quantification along with statistical analysis. How often were the experiments in Fig 5 F and G repeated?
Response: Yes, we did repeat experiments and 4 mice per group were used for the data analysis. We now included statistical analysis **in Figure 5G and 5I**. Our results showed that c-fos expression was significantly increased in ipsilateral side (Ipsi.) compared with contralateral side (Contra.) in ReaChR mice at 2hr after light stimulation, or compared with that in control mice (**Figure 5G**, n=4 mice/group, ****P < 0.0001. Two-way ANOVA with multi comparisons). Also, PKCα was co-localized with NeuN$^+$ neurons but not with Iba1$^+$ microglia in the spinal dorsal horn at 3 days after light stimulation in ReaChR mice (**Figure 5I**, n=4 mice/group, ****P <

0.0001. Unpaired Student’s t-test).

4. No n values are given for Fig. 6A or for Fig 7. This is also true for some of the supplemental data. Each legend should clearly state the numbers of repetitions.
Response: We now added n values for Figure 6A and Figure 7 in figure legends, and Supplementary Figure 1, 2, 4, and 5 (**now Supplementary Figure 1, 2, 10 and 12**).

5. The discussion does not mention recent work on the role of Pannexins in microglial pain signaling, which is pertinent here (see work from Trang lab in Nature Medicine and Science Advances). Does the opto-activation of microglia trigger pannexin pore formation? Does probenecid block the opto induced effects observed here?
Response: We are grateful to the reviewer’s insightful comments. To address questions on the role of pannexin-1, we performed additional experiment using probenecid as suggested. We found that single systemic injection of probenecid (100 mg/kg) did not prevent the progressive reduction in mechanical threshold or reversed the established mechanical allodynia after optogenetic activation of spinal microglia. These results suggest that optogenetic activation of microglia may not trigger pannexin pore formation. Nevertheless, we included the results in the **supplementary Figure 11** and discuss the results together with recent studies (Burma et al., 2017; Mousseau et al., 2018) on the role of pannexin-1 in microglia pain signaling (**please see paragraph 2, page 3 and please see paragraph 3, page 9**).

Responses to Reviewer 3:

This is an elegantly designed and comprehensive study which reveals novel aspects on the role of spinal microglial activation, achieved via optogenetic stimulation, and its consequence on inducing pain sensitization. The authors show that a 30-minute light stimulation, which specifically depolarizes spinal microglia, triggers chronic pain hypersensitivity lasting for up to 8 days. Light stimulation leads to the adoption of an activated microglial phenotype characterized by a deramified morphology, up-regulation of cellular activation markers and the release of IL-1beta. As a central pro-inflammatory mediator, IL-1beta induces an increase in nociceptive transmission as shown by larger C-fiber-evoked field potentials which is abolished by IL-1 receptor antagonism. Remarkably, blockade of IL-1beta signaling not only prevents the enhanced nociceptive neuronal activity, but also completely abrogates pain hypersensitivity. In summary, by using a technically advanced approach this work demonstrates that a transient optogenetic depolarization of spinal microglia is a sufficient stimulus to rapidly activate microglia and trigger long-lasting pain hypersensitivity in mice via an IL-1beta-dependent mechanism.
Response: We thank the reviewer’s positive comments on our study.

1. Despite the pivotal role of IL-1beta signaling, it remains mechanistically unclear how ReaChR2 activates microglia, especially concerning the role of [Ca2+]i. ReaChR2 stimulation broadly mimics the activity of non-selective cation channels by inducing cell depolarization via non-selective cation fluxes across the membrane. However, as the authors discuss, a strong and sustained ChR2 activation may also elevate [Ca2+]i and is expected to decrease intracellular [K+]; key signaling events that are mechanistically associated with microglial activation, e.g. by regulating inflammasome assembly (see https://pubmed.ncbi.nlm.nih.gov/30755589/). Furthermore, sustained ChR2 activation is known to increase extracellular [K+] (https://pubmed.ncbi.nlm.nih.gov/31116972/), which may indirectly affect neuronal activity in addition to IL-1beta. To better understand the mechanisms by which ReaChR2 activates microglia, changes of [Ca2+]i following light stimulation should be examined (although

the discussion implies that this has been tested, data is not shown), as well as the potential role of Ca2+ in promoting ReaChR2-induced release of IL-1beta (related to Fig. 6, e.g. by omitting Ca2+ from the ACSF). Likewise, potential changes in extracellular K+ should be examined after 30 minutes of ReaChR2 stimulation. Although difficult to address experimentally, the potential impact of a decreased [K+]i following ChR2 stimulation (compared to changes in membrane voltage) on inducing microglial activation and IL-1beta release should at least be discussed.
Response: We appreciate the excellent advice on testing the potential consequences of Ca^{2+} elevation after ReaChR activation. Accordingly, we performed additional experiments to chelate extracelluar Ca^{2+} using EGTA (5 mM) from primary microglia culture media during light stimulation *in vitro* and then tested ReaChR2-induced release of IL-1β. We found that IL-1β release was blocked by chelating Ca^{2+} at 1, 6, and 24hr after light stimulation. Our results suggest that microglial ReaChR activation triggers Ca^{2+} elevation in promoting the release of IL-1β. Similarly, it is suggested that IL-1β release via P2X7 is also triggered by increased intracellular Ca^{2+} (Gudipaty et al., 2003; Andrei et al., 2004). Activation of ReaChR in neurons is reported to induce Ca^{2+} elevation (Chen et al., 2019), which may be directly through ReaChR itself and/or indirectly through voltage-gated Ca^{2+} channels. Future studies are needed to use microglial Ca^{2+} imaging to study ReaChR activation (Umpierre et al., 2020). We included additional data in the **Figure 6H** (**Please also see paragraph 3, page 9**).

Yes, we agree with the potential importance of the intracellular or extracellular K^+ change after microglia ReaChR activation. Also, it is reported that ChR2-mediated extracellular K^+ elevations may also affect the excitability of neurons (Octeau et al., 2019), while ChR2-mediated intracellular K^+ reduction may be critical for proinflammatory cytokines like IL-1β (Pétrilli et al., 2007). We have included these points in the discussion (**Please see paragraph 2, page 13**).

2. Mechanistically, the authors show that light-evoked microglial activation rapidly enhances nociceptive transmission by augmenting C-fiber responses (Fig. 5C), while blockade of IL-1beta signaling prevents these effects and completely abrogates pain hypersensitivity (Fig. 7). However, as shown in Fig. 5C, the increase in C-fiber responsiveness is only transient and lasts only for ~ 90 min before returning to baseline after 2h. How do the authors explain the transient nature of an increased neuronal activity (i.e. C-fiber response) with the chronicity of the induced pain phenotype (mechanical allodynia and thermal hyperalgesia), which lasts for more than 7 days (Fig. 3G, H)? It seems that the initial IL-1beta dependent rise in neuronal activity acts as a trigger to induce long-lasting cellular changes (such as the observed up-regulation of PKC-alpha) which then give rise to sustained pain hypersensitivity. Additional experiments are required to gain further mechanistic insights into how PKC-alpha produces long-term changes in neuronal transmission related to the observed chronic pain phenotype.
Response: We thank the reviewer for the great questions. Indeed, we found that optogenetic stimulation acutely increased synaptic transmission but the pain behaviors lasted for days. One explanation for the results is that optogenetic stimulation induces microglia activation, which is maintained for several days as shown in **Figure 4**. Activated microglia may chronically sensitize spinal neurons through biochemical cascade, such as PKCα signaling, that contribute to chronic pain hypersensitivity. Consistent with this idea, recent studies have reported that microglia are crucial for synaptic plasticity between C-fibers and spinal neurons that underlying chronic pain (Clark et al., 2015; Zhou et al., 2019).

As summarized by the reviewer, we believe that the initial IL-1beta release from microglia acts as a trigger to induce long-lasting neuronal changes including up-regulation of PKCα. To address the reviewer's question on how PKCα participate in chronic pain, we performed additional experiments to pharmacologically inhibit PKCα, which known for blocking calcium-dependent PKC translocation and function (Ron et al., 1995), in ReaChR-induced pain. Interestingly, we observed that a single intrathecal injection of PKCα inhibitor peptide (Cayman,

Cat# 17478, 10 μM, i.t) before optic stimulation prevented the progressive mechanical threshold reduction. Accordingly, our results indicate that PKCα is the key player for maintaining chronic pain responses after optogenetic stimulation of microglia. We did not test how neuronal PKCα may be involved in maintaining chronic pain, but previous studies have suggested that PKCα activation leads to GluR2 internalization in dorsal horn neuron, which increases Ca^{2+} permeability and promotes chronic pain (Park et al., 2009). We included additional data in the **Supplementary Figure 8** and discussed the possible mechanism underlying PKCα in chronic pain (**Please see paragraph 1, page 13**).

3. A striking outcome of this work is the rapid onset (and longevity) of pain hypersensitivity (<1h up to 8 days) after a just 30-minute light-induced depolarization of spinal microglia (Fig. 3G, H). However, to better correlate the time course of the behavioral (and neuronal) effects with early signs of microglial changes, it would be essential to investigate this also at 30 min - 1 h after light stimulation (by examining microglial morphological changes and up-regulation of classical microglial activation markers in addition to Kv1.3, such as CD68 or IBA-1, related to Fig. 4). Moreover, microglial activation and morphology should also be investigated after the pain symptoms have recovered at 9 days post stimulation.

Response: Yes, we agree with the reviewer to further investigate microglial activation at early and late time points after light stimulation. We performed several lines of additional experiments to address the concern:

(1) To examine the morphological changes, we performed additional sholl analysis at 1hr, 7, and 9 days after light stimulation. Our results showed that light stimulation significantly induced shorter processes and less complexity at 1-5 days after light stimulation compared to controls (**Figure 4D-E**). However, morphological changes were largely recovered at 7-9 days **(Supplementary Figure 5).** The additional analysis was included in **Supplementary Figure 5** (**Please also see paragraph 1, page 7**).

(2) To examine the microglial activation, we attempted to use representative microglia activation marker p-p38 in neuropathic pain (Ji and Suter, 2007). However, we found that immunostaining with p-p38 antibody (4511S, RRID:AB_2139682, CST) did not work well in our hands for immunostaining in mice. We then additionally performed WB analysis to detect p-p38 expression. We found that p-p38 was increased at 1 day and 3 days after light stimulation. We included additional data in the **Supplementary Figure 7** (**Please also see paragraph 1, page 7**).

(3) Finally, we examined Kv1.3 expression in $Iba1^{+}$ spinal microglia at 30 min, 1hr, 1D, 3D, 5D, and 9D after light stimulation. We found that Kv1.3 was gradually increased in $Iba1^{+}$ cells at 30 min, 1hr, 1D, 3D, 5D days but not at 9 days following optogenetic stimulation. Interestingly, Kv1.3 expression was upregulated in microglial process than the cell body during the early time window (30 min-1 hr), but the upregulation was significant in both processes and the cell body at 1- 3 days after light stimulation. We included additional data in the **Supplementary Figure 6** (**Please also see paragraph 1, page 7**).

4. The resting membrane potential of patch-clamped spinal microglia in voltage clamp (by using voltage steps) shows a reversal potential of ~ -60 mV (Fig. 2C), while in current clamp mode (Fig. 2F) the cells seem to be much more depolarized at ~ -20 mV (Fig. 2F). What is the average resting membrane potential of spinal microglia and how is the difference in Vrest between the two recordings modes to be explained? In addition, what is the rationale for using an intracellular patch solution with a pH set to 7.5? (rather than 7.1 - 7.2, which is the physiological resting intracellular pH of cells, https://pubmed.ncbi.nlm.nih.gov/19997129/)

Response: We apologize that there was a typo in the description of the intracellular patch solution. An intracellular patch solution at pH 7.2 was actually used. We corrected the error in the revision (**Please see paragraph 2, page 18**).

In the current clamp mode in our microglia recordings, we found the resting potential of microglia is around -20 mV. In the voltage clamp mode, the baseline current of microglia is very small and thus the reversal potential may not be accurate (Wu et al., 2007). In addition, the holding potential of the recording is at -60 mV, which was used for maintaining microglia with a good shape during the voltage steps stimulation (Schilling and Eder, 2007). The small leak at -60 mV was corrected as the baseline and thus the reversal potential is close to -60 mV.

5. How do the authors explain the comparatively rapid release (<1 h) of IL-1beta in response to light stimulation in the absence of a priming stimulus, which normally is required to trigger inflammasome assembly and activation of caspase-1 (Fig. 6)?
Response: Yes, it is important to understand whether inflammasome is involved after optogenetic stimulation. The kinetics and mechanism of IL-1β release from microglia or other cells appear to depend on various pathological conditions (Sanz and Di Virgilio, 2000; MacKenzie et al., 2001; Sun et al., 2019). Mature IL-1β is rapidly secreted (even within 25 min) from the cell following caspase-1-dependent processing of pro-IL-1β (Brough and Rothwell, 2007). Also, it is well known that caspase-1 activity is enhanced by P2X7 and pannexin-1 stimulation (Pelegrin and Surprenant, 2006). Particularly, secretion IL-1β via P2X7 is triggered by K^+ efflux and increased intracellular Ca^{2+} (Gudipaty et al., 2003; Andrei et al., 2004). Similar to the reviewer's opinion, priming stimulus like inflammasome assembly proceeds to cleave the cytokine precursors pro- IL-1β into mature IL-1β (Yang et al., 2019). Therefore, we performed additional experiment to observe priming step by activation of NLRP3 inflammasome and caspase-1 using WB analysis. We found that NLRP3 and caspase-1 was increased from 1hr to 3 days after light stimulation. Accordingly, we believe that ReaChR activation may also act as priming stimulus in addition to trigger IL-1β release after optogenetic stimulation. We included additional data in the **Figure 6C-D** (**Please also see paragraph 2, page 9**).

6. The up-regulation of c-fos (Fig. 5F) appears to occur also at the non-treated contralateral side. Please provide more convincing immunohistochemical or other evidence to better support this claim.
Response: Yes, we confirmed c-fos expression in a small number of c-fos$^+$ cells in the contralateral side in four more independent experiments. Nevertheless, the increase of c-fos expression is clearly more in the ipsilateral side compared with contralateral side after light stimulation. We have now included the statistical results in **Figure 5G.** Although further study is needed to understand the mechanism underlying mirror activation in the opposite side in microglial optogenetic pain model, similar phenomenon was observed after peripheral never injury (Rashid and Ueda, 2005; Gao and Ji, 2009).

7. Despite the recently emerging controversial views on the role of P2X4-induced expression of microglial BDNF to mediate neuropathic pain by changing neuronal chloride gradients (see e.g. https://pubmed.ncbi.nlm.nih.gov/16355225/, https://pubmed.ncbi.nlm.nih.gov/31315039/, https://pubmed.ncbi.nlm.nih.gov/24089642/), I wonder how the proposed IL-1beta-dependent mechanism fits in with the prevailing BDNF/chloride story. Did you test whether ChR2 stimulation mimics some aspects of P2X4 signaling and, apart from IL-1beta, also causes the release of other microglial mediators such as BDNF or TNF-alpha? Mechanistic parallels and differences between the P2X4/BDNF- and ChR2/IL1-beta-dependent pathways on inducing neuropathic pain should also be discussed in more detail.
Response: We thank the reviewer for the great question and suggestion. To address the concerns, we performed additional experiments using qRT-PCR to examine the other microglial

mediators such as BDNF, IL-1β, TNFα, IL6, and CCL2. Our results showed that the IL-1β was dramatically increased at 1 day and 3 days after microglia optogenetic stimulation compared with the control group. Also, BDNF expression was significantly increased at 3 days. To further test the BDNF release following optogenetic stimulation, we also performed ELISA assay to detect BDNF in primary microglia expressing ReaChR *in vitro*. Interestingly, we found that there are no significant changes in BDNF release following optogenetic stimulation. Therefore, this result indicates that light stimulation of ReaChR-expressing microglia release IL-1β preferentially compared to other mediators. We have included the results in **Supplementary Figure 9** (**Please also see paragraph 3, page 8**).

Responses to Reviewer 4:

This is a nice study demonstrating optogenetic activation of spinal microglia to induce pain behaviors and increases in C-fiber evoked responses. The study is well controlled and the results are of broad interest. There are several issues to be addressed.
Response: We appreciate the reviewer's positive feedback.

1. The investigation of the activation state of microglia is a bit thin. The morphological analysis is appropriate, but there are not clear links to other endpoints in this study (e.g., IL-1b). What about activation of signaling cascades, such as MAP kinases and NFkB?
Response: We thank the reviewer for the great suggestions. We performed additional experiments using WB analysis to examine the signaling cascades such as MAP kinase and NFkB pathways. We observed that the p-38 and p-Erk was dramatically increased at 1 day and 3 days after microglia optogenetic stimulation compared with control group. However, we did not observe either the increase of p-JNK or p-P65 in NF-κB pathway. We have included the results in **Supplementary Figure 7** (**Please also see paragraph 1, page 7**).

2. The authors could also consider transcriptional profiling of opto-activated microglia to provide a more comprehensive assessment of the activation state.
Response: This is a great suggestion. Yes, we are planning more comprehensive transcriptional profiling of optogenetically activated microglia, such as RNAseq experiments for future study. Here, to further characterize microglial activation by optogenetic stimulation, we added several lines of evidence in the revision: (1) We performed additional experiments using qRT-PCR to examine the other microglial mediators such as BDNF, IL-1β, TNFα, IL6, and CCL2. Our results showed that the IL-1β was dramatically increased at 1 day and 3 days after microglia optogenetic stimulation compared with the control group (**Supplementary Figure 9A-E**); (2) Using WB analysis, we examined the expression of NLRP3 Inflammasome and caspase-1. We found that NLRP3 and caspase-1 was increased from 1hr to 3 days after light stimulation (**Figure 6C-D**); (3) We performed additional experiments using Ki67 immunostaining to study microglial proliferation after optogenetic stimulation. We found increased proliferation of $Iba1^+$ microglia at 3 days, which was reduced at 5-7 days in ReaChR mice after optogenetic stimulation (**Figure 4C** and **Supplementary Figure 4A-B**). We have included these additional results in the revision (**Please also see paragraph 1, page 7 and paragraph 1-2, page 9**).

3. It is interesting that acute IL-1ra enduringly prevents nociceptive hypersensitivity induced by ReaChR stimulation. The data indicate that ReaChR stimulation induces acute release of IL-1b that induces secondary processes, supported by experiment showing that IL-1ra administered 3 days after light stimulation does not reduce PKCα levels in spinal neurons. This should be confirmed behaviorally.

Response: We thank the reviewer for the great comment. Yes, we performed additional pain behavior tests after PKCα inhibitor treatment. Interestingly, we observed that single injection of PKCα inhibitor peptide (Cayman, Cat# 17478, 10 μM, i.t) before optogenetic stimulation attenuated light-induced chronic pain. PKCα inhibitor peptide are known for blocking calcium-dependent PKC translocation and function (Ron et al., 1995). These results indicate that PKCα is a key player in mediating pain hypersensitivity after microglial ReaChR activation. We have included the results in **Supplementary Figure 8** (**Please also see paragraph 2, page 8**).

4. The cellular mechanism(s) should be investigated. For example, does calcium influx activate NLRP3 inflammasomes to cleave constitutive pro-IL-1b?
Response: We agree with the reviewer to investigate further mechanisms underlying ReaChR-dependent IL1β production. As suggested, to test whether NLRP3 participates in IL-1β production following optogenetic stimulation, we performed WB analysis in the spinal cord and found that both NLRP3 and capspase-1 was increased after light stimulation in ReaChR mice compared with control (**Figure 6C-D**). In addition, we examined the role of extracellular Ca^{2+} in IL1β production in cultured microglia. When we chelated Ca^{2+} using EGTA (5 mM) during light stimulation *in vitro*, we observed IL-1β release was completely blocked (**Figure 6H**). Together, these results suggest that Ca^{2+} is directly implicated as priming stimulus as well as promoting IL-1β release by microglial ReaChR activation. We have included the results in **Figure 6** (**Please also see paragraph 2-3, page 9**).

5. Is autocrine signaling of IL-1b at microglia necessary to establish the persistent nociceptive hypersensitivity, or is it maintained by the neurons?
Response: We believe that the hypersensitivity is maintained by neurons. The release of IL-1β from microglia after optogenetic stimulation triggered the neuronal responses including increased synaptic transmission (**Figure 5C-D**) and upregulation of PKCα (**Figure 5H-I**). Consistently, we observed that a single intrathecal injection of recombinant IL-1β protein induces both mechanical and thermal pain responses lasting for multiple hours (**Supplementary Figure 10A-B**). However, exogenous IL-1β was able to upregulate endogenous IL-1β expression in microglia (**Supplementary Figure 10C-D**), suggesting an amplified response for microglial IL-1β signaling.

6. The sex of the mice should be included in the title and abstract.
Response: Thanks for the suggestion. Yes, we observed nociceptive hypersensitivity in both male and female mice after light stimulation (**Supplementary Figure 3**). We now included the sex of the mice in the abstract.

References

Andrei C, Margiocco P, Poggi A, Lotti LV, Torrisi MR, Rubartelli A (2004) Phospholipases C and A2 control lysosome-mediated IL-1 beta secretion: Implications for inflammatory processes. Proc Natl Acad Sci U S A 101:9745-9750.

Brough D, Rothwell NJ (2007) Caspase-1-dependent processing of pro-interleukin-1beta is cytosolic and precedes cell death. J Cell Sci 120:772-781.

Burma NE, Bonin RP, Leduc-Pessah H, Baimel C, Cairncross ZF, Mousseau M, Shankara JV, Stemkowski PL, Baimoukhametova D, Bains JS, Antle MC, Zamponi GW, Cahill CM, Borgland SL, De Koninck Y, Trang T (2017) Blocking microglial pannexin-1 channels alleviates morphine withdrawal in rodents. Nat Med 23:355-360.

Chen IW, Ronzitti E, Lee BR, Daigle TL, Dalkara D, Zeng H, Emiliani V, Papagiakoumou E (2019) Submillisecond Two-Photon Optogenetics with Temporally Focused Patterned Light. J Neurosci 39:3484-3497.
Clark AK, Gruber-Schoffnegger D, Drdla-Schutting R, Gerhold KJ, Malcangio M, Sandkühler J (2015) Selective activation of microglia facilitates synaptic strength. J Neurosci 35:4552-4570.
Gao YJ, Ji RR (2009) c-Fos and pERK, which is a better marker for neuronal activation and central sensitization after noxious stimulation and tissue injury? Open Pain J 2:11-17.
Gudipaty L, Munetz J, Verhoef PA, Dubyak GR (2003) Essential role for Ca2+ in regulation of IL-1beta secretion by P2X7 nucleotide receptor in monocytes, macrophages, and HEK-293 cells. Am J Physiol Cell Physiol 285:C286-299.
Ji RR, Suter MR (2007) p38 MAPK, microglial signaling, and neuropathic pain. Mol Pain 3:33.
Lin JY, Knutsen PM, Muller A, Kleinfeld D, Tsien RY (2013) ReaChR: a red-shifted variant of channelrhodopsin enables deep transcranial optogenetic excitation. Nat Neurosci 16:1499-1508.
MacKenzie A, Wilson HL, Kiss-Toth E, Dower SK, North RA, Surprenant A (2001) Rapid secretion of interleukin-1beta by microvesicle shedding. Immunity 15:825-835.
Mousseau M, Burma NE, Lee KY, Leduc-Pessah H, Kwok CHT, Reid AR, O'Brien M, Sagalajev B, Stratton JA, Patrick N, Stemkowski PL, Biernaskie J, Zamponi GW, Salo P, McDougall JJ, Prescott SA, Matyas JR, Trang T (2018) Microglial pannexin-1 channel activation is a spinal determinant of joint pain. Sci Adv 4:eaas9846.
Octeau JC, Gangwani MR, Allam SL, Tran D, Huang S, Hoang-Trong TM, Golshani P, Rumbell TH, Kozloski JR, Khakh BS (2019) Transient, Consequential Increases in Extracellular Potassium Ions Accompany Channelrhodopsin2 Excitation. Cell Rep 27:2249-2261.e2247.
Park JS, Voitenko N, Petralia RS, Guan X, Xu JT, Steinberg JP, Takamiya K, Sotnik A, Kopach O, Huganir RL, Tao YX (2009) Persistent inflammation induces GluR2 internalization via NMDA receptor-triggered PKC activation in dorsal horn neurons. J Neurosci 29:3206-3219.
Pelegrin P, Surprenant A (2006) Pannexin-1 mediates large pore formation and interleukin-1beta release by the ATP-gated P2X7 receptor. EMBO J 25:5071-5082.
Pétrilli V, Papin S, Dostert C, Mayor A, Martinon F, Tschopp J (2007) Activation of the NALP3 inflammasome is triggered by low intracellular potassium concentration. Cell Death Differ 14:1583-1589.
Rashid MH, Ueda H (2005) Pre-injury administration of morphine prevents development of neuropathic hyperalgesia through activation of descending monoaminergic mechanisms in the spinal cord in mice. Mol Pain 1:19.
Ron D, Luo J, Mochly-Rosen D (1995) C2 region-derived peptides inhibit translocation and function of beta protein kinase C in vivo. J Biol Chem 270:24180-24187.
Sanz JM, Di Virgilio F (2000) Kinetics and mechanism of ATP-dependent IL-1 beta release from microglial cells. J Immunol 164:4893-4898.
Schilling T, Eder C (2007) Ion channel expression in resting and activated microglia of hippocampal slices from juvenile mice. Brain Res 1186:21-28.
Sun L, Ma W, Gao W, Xing Y, Chen L, Xia Z, Zhang Z, Dai Z (2019) Propofol directly induces caspase-1-dependent macrophage pyroptosis through the NLRP3-ASC inflammasome. Cell Death Dis 10:542.
Umpierre AD, Bystrom LL, Ying Y, Liu YU, Worrell G, Wu LJ (2020) Microglial calcium signaling is attuned to neuronal activity in awake mice. Elife 9.
Wu LJ, Vadakkan KI, Zhuo M (2007) ATP-induced chemotaxis of microglial processes requires P2Y receptor-activated initiation of outward potassium currents. Glia 55:810-821.

Yang Y, Wang H, Kouadir M, Song H, Shi F (2019) Recent advances in the mechanisms of NLRP3 inflammasome activation and its inhibitors. Cell Death Dis 10:128.

Zhou LJ, Peng J, Xu YN, Zeng WJ, Zhang J, Wei X, Mai CL, Lin ZJ, Liu Y, Murugan M, Eyo UB, Umpierre AD, Xin WJ, Chen T, Li M, Wang H, Richardson JR, Tan Z, Liu XG, Wu LJ (2019) Microglia Are Indispensable for Synaptic Plasticity in the Spinal Dorsal Horn and Chronic Pain. Cell Rep 27:3844-3859 e3846.
