# Supplementary material for: Optogenetic activation of spinal microglia triggers chronic pain in mice
Source: PLoS Biol. 2021 Mar 19;19(3):e3001154. doi: 10.1371/journal.pbio.3001154 (PMC8011727; doi:10.1371/journal.pbio.3001154)
Supplement: Attachment — Submitted filename: Response to reviewers 20210217.pdf [file pbio.3001154.s016.pdf]

Point-to-point response to the reviewers' comments (PBIOLOGY-D-20-02660R2)

Responses to Reviewer 4:

The authors have done an excellent job in addressing comments from all reviewers, having added substantially more data to the manuscript.
Response: We appreciate the reviewer's positive feedback.

I have only a minor comment regarding this sentence from the introduction: "However, it is still unclear whether direct microglial activation is sufficient to trigger pain hypersensitivity." This has been partly addressed using DREADDs, and should be acknowledged (https://pubmed.ncbi.nlm.nih.gov/29530713/)
Response: We thank the reviewer for pointing out this. We have acknowledged recent studies using chemogenetic manipulations of microglia in the revision (Please see 1st paragraph, page 3).
